# Artificial intelligence in mapping nursing diagnoses, interventions, and outcomes for diabetes management

**DOI:** 10.17533/udea.iee.v44n1e07

**Published:** 2026-03-29

**Authors:** Beatriz Barboza Fernandes, Rodrigo de Araujo Marques, Rosane Barreto Cardoso, Rejane Prado dos Santos, Thaíssa Felix Affonso

**Affiliations:** 1 Nurse, master's student. Email: beatrizbarbozafernandes@gmail.com https://orcid.org/0009-0001-7789-1548 Universidade Federal do Rio de Janeiro Brazil beatrizbarbozafernandes@gmail.com; 2 Nurse. Email: rodrigo.armarques@gmail.com https://orcid.org/0009-0002-3393-5155 Universidade Federal do Rio de Janeiro Brazil rodrigo.armarques@gmail.com; 3 Nurse, Ph.D. Email: rosane.bcardoso@gmail.com Corresponding author. https://orcid.org/0000-0001-8052-8697 Universidade Federal do Rio de Janeiro Brazil rosane.bcardoso@gmail.com; 4 Nurse, master's student. Email: rejaneprado@hucff.ufrj.br https://orcid.org/0000-0002-9898-4399 Universidade Federal do Rio de Janeiro Brazil rejaneprado@hucff.ufrj.br; 5 Nurse, master's student. Email: thaifelixa@gmail.com https://orcid.org/0009-0001-6719-8643 Universidade Federal do Rio de Janeiro Brazil thaifelixa@gmail.com; 6 Federal University of Rio de Janeiro, Rio de Janeiro, Brazil Universidade Federal do Rio de Janeiro Federal University of Rio de Janeiro Rio de Janeiro Brazil

**Keywords:** diabetes mellitus type 2, nursing process, nursing diagnosis, treatment outcome, standardized nursing terminology, nursing care, artificial intelligence, cross-mapping., diabetes mellitus tipo 2, proceso de enfemería, diagnóstico de enfermería, resultado del tratamento, terminología normalizada de enfermeira, atención de enfermeira, inteligencia artificial, mapeamento cruzado., diabetes mellitus tipo 2, processo de enfermagem, diagnóstico de enfermagem, resultado do tratamento, terminologia padronizada em enfermagem, cuidados de enfermagem, inteligência artificial, mapeamento cruzado.

## Abstract

**Objective.:**

To map nursing diagnoses, nursing outcomes, and nursing interventions based on the clinical indicators described in the *Type 2 Diabetes Diagnosis and Management Manual*, using artificial intelligence (AI) (GPT-4®).

**Methods.:**

Descriptive study with adapted cross-mapping. GPT-4® was applied with a structured prompt to identify clinical indicators in the manual and correlate them with nursing classifications.

**Results.:**

AI identified 43 clinical indicators, and after manual review, 30 were confirmed, with 23 overlapping between methods. From these, 30 nursing diagnoses, 30 expected outcomes, and 30 interventions were mapped. Manual mapping consolidated 15 nursing diagnoses, 15 outcomes, and 15 interventions.

**Conclusion.:**

AI proved effective in expediting and standardizing cross-mapping in nursing. However, human clinical judgment was indispensable to validate and adjust inconsistencies, capturing nuances not identified by AI. The integration of AI with clinical reasoning can strengthen care systematization, support evidence-based protocols, and improve outcomes in patients with diabetes.

## Resumen

**Objetivo.:**

Mapear diagnósticos, resultados e intervenciones de enfermería a partir de los indicadores clínicos descritos en el Manual Diagnóstico y Manejo de la diabetes tipo 2, utilizando inteligencia artificial (IA) (GPT-4®).

**Métodos.:**

Investigación descriptiva con mapeo cruzado adaptado. Se utilizó GPT-4® con un prompt estructurado para identificar indicadores clínicos en el manual y correlacionarlos con las clasificaciones de enfermería.

**Resultados.:**

La IA identificó 43 indicadores clínicos y, después de una revisión manual, se confirmaron 30, con 23 coincidencias entre los dos métodos. Se mapearon 30 diagnósticos, 30 resultados esperados y 30 intervenciones. En el mapeo manual se consolidaron 15 diagnósticos, 15 resultados esperados y 15 intervenciones.

**Conclusión.:**

La IA demostró ser eficaz para agilizar y estandarizar el mapeo cruzado en enfermería. Sin embargo, el juicio clínico humano fue indispensable para validar y ajustar inconsistencias, captando matices no identificados por la IA. La integración entre la IA y el razonamiento clínico puede fortalecer la sistematización de la atención, respaldar protocolos basados en la evidencia y mejorar los resultados en pacientes con diabetes.

## Resumo

**Objetivo.:**

Mapear diagnósticos de enfermagem, resultados de enfermagem e intervenções de enfermagem a partir dos indicadores clínicos descritos no manual de Diagnóstico e manejo do diabetes tipo 2, utilizando inteligência artificial (IA) (GPT-4®).

**Métodos.:**

Pesquisa descritiva com mapeamento cruzado adaptado. Foi utilizado GPT-4® com prompt estruturado para identificar indicadores clínicos no manual e correlacioná-los às classificações de enfermagem.

**Resultados.:**

A IA identificou 43 indicadores clínicos e, após revisão manual, 30 foram confirmados, com 23 coincidindo entre os métodos. Foram mapeados 30 diagnósticos, 30 resultados esperados e 30 intervenções. No mapeamento manual, ficaram consolidados 15 diagnósticos, 15 resultados esperados e 15 intervenções.

**Conclusão.:**

A IA mostrou-se eficaz para agilizar e padronizar o mapeamento cruzado em enfermagem. Entretanto, o julgamento clínico humano foi indispensável para validar e ajustar inconsistências, captando nuances não identificadas pela IA. A integração entre IA e raciocínio clínico pode fortalecer a sistematização do cuidado, apoiar protocolos baseados em evidências e aprimorar desfechos em pacientes com diabetes.

## Introduction

Diabetes mellitus currently represents one of the greatest challenges to global public health, with a growing impact especially in low- and middle-income countries. According to the World Health Organization (WHO), the number of people living with diabetes increased from 200 million in 1990 to 830 million in 2022, with more than half of these people not using medication to control the disease due to low treatment coverage.^1^ This reality reinforces the need for care strategies that promote early diagnosis, continuous monitoring, and adherence to treatment, especially in primary health care. In this context, nursing professionals play a key role, as they are directly involved in clinical monitoring, support for self-care, and the prevention of complications associated with the disease. The Nursing Process (NP)^2^ is an essential tool for structuring and qualifying this care, especially when supported by standardized terminologies, such as the North American Nursing Diagnosis Association-International (NANDA-I), the Nursing Outcomes Classification (NOC), and the Nursing Interventions Classification (NIC). The use of these classifications contributes to the standardization of records, interprofessional communication, and the evaluation of health outcomes.^3^

The Pan American Health Organization (PAHO), with the manual Diagnosis and Management of Type 2 Diabetes (HEARTS-D),^4^ provides clinical guidelines that guide safe and standardized practices in disease management. However, there is still a gap regarding the systematic translation of the manual's clinical indicators into nursing diagnoses, outcomes, and interventions, which could enhance its applicability in clinical practice. The use of standardized terminology in care protocols is relevant in the context of diabetes, as the disease presents specific challenges in terms of diagnosis and treatment. Standardization allows different professionals and sectors to clearly understand nursing diagnoses, outcomes, and interventions, promoting a more integrated and effective approach.^5^

Studies indicate that the use of Artificial Intelligence (AI) can optimize the cross-mapping of diagnoses, outcomes, and nursing interventions, making the process faster and more accurate. Advanced AI models, such as GPT-4®, have shown the ability to analyze large volumes of data and identify clinical patterns, facilitating decision-making in nursing.^6^^,^^7^ AI is designed to simulate human intelligence, being capable of performing tasks such as pattern recognition, clinical data analysis, and decision-making support.^7^ AI can automate repetitive processes, freeing professionals to focus on more complex activities and ensuring greater accuracy in identifying nursing diagnoses.^6^ Given the specificity of diabetes management, this study aims to map nursing diagnoses, nursing outcomes, and nursing interventions based on the clinical indicators described in the PAHO HEARTS-D manual, using AI. 

## Methodology 

### Type of Study

This is a descriptive study that used the adapted cross-mapping technique, as described by Lucena and Barros,^8^ to correlate nursing diagnoses (ND), based on the NANDA-I classification, nursing outcomes (NO), according to the NOC classification, and nursing interventions (NI), according to the NIC classification, considering the clinical indicators of type 2 diabetes mellitus established in the PAHO HEARTS-D manual. The cross-mapping method is an approach that allows comparing non-standardized clinical data with formal nursing classifications, enabling the identification of semantic and clinical correspondences.^8^ This process promotes the standardization of documentation, enhances nurses' clinical reasoning, and facilitates evidence-based decision-making. To optimize the identification of ND, NO, and NI, GPT-4® AI was employed, an advanced language model that enables the analysis of large volumes of data, the interpretation of clinical patterns, and the systematic structuring of information.

### Methodological Procedure

In order to ensure the accuracy of AI in the analysis, a structured prompt was developed based on prompt engineering principles,^9^ including: (i) Clear objective: guiding the AI to perform cross-mapping of clinical indicators with the NANDA-I, NOC, and NIC classifications; (ii) Progressive steps: hierarchical organization that includes identification of indicators, diagnostic correlation, expected outcomes, and interventions; (iii) Standardization of responses: inclusion of numbered rules to guide the model in extracting relevant information and formatting it correctly; (iv) Limited sources: restricting the AI to use only official manuals and classifications, avoiding irrelevant or generic information; and (v) Minimization of ambiguities: detailed structuring to reduce uncertainties and ensure the accuracy of clinical matches. This prompt was adapted to follow the cross-mapping guidelines described by Lucena and Barros.^8^ See Table 1.

**Table 1:** Structured prompt for mapping nursing diagnoses, outcomes, and interventions

1. Identification of Clinical Indicators Based on the document “Diagnosis and Management of Type 2 Diabetes (HEARTS-D),” all clinical indicators should be identified, such as: Defining Characteristics: Signs, symptoms, and observable clinical manifestations of diabetes. Risk Factors: Conditions that predispose individuals to the development or worsening of diabetes and its complications. Populations at Risk: Vulnerable groups due to epidemiological, sociodemographic, and environmental exposure factors. Associated Conditions: Diagnostic or therapeutic procedures, medical diagnoses, use of devices or pharmaceutical preparations. 2. Cross-Mapping for Nursing Diagnoses (NANDA-I) Based on the clinical indicators: (INSERT INDICATORS), a cross-mapping must be performed to associate them with nursing diagnoses (NANDA-I), 13^th^ edition, following the guidelines below: Link all clinical indicators with the corresponding elements of NANDA-I diagnoses. Prioritize the semantic analysis of the terms, avoiding mechanical associations based solely on keywords. Allow the splitting of the same clinical indicator for more than one NANDA-I diagnosis when there is concept overlap. In the absence of an exact match, select the diagnosis closest to the clinical situation. Ensure that all nursing diagnoses used are listed in the appendix “NANDA-I Nursing Diagnoses List.” 3. Association with Nursing Outcomes (NOC) For each identified nursing diagnosis, at least one expected outcome (NOC) from the 7^th^ edition must be associated, allowing objective measurement of the patient’s progress. The rules for selection are: Each nursing diagnosis must be linked to at least one NOC outcome described in the annex “Nursing Outcomes Classification (NOC).” Select measurable clinical indicators within the NOC scales, considering those that best reflect the patient’s condition. Select measurable clinical indicators within the NOC scales, considering those that best reflect the patient’s condition. Ensure that all selected outcomes are described in the appendix “List of Nursing Outcomes (NOC).” 4. Mapping for Nursing Interventions (NIC) For each combination of nursing diagnosis (NANDA-I) + expected outcome (NOC), appropriate nursing interventions (NIC), 7^th^ edition, must be selected, following these guidelines: Each intervention must be directly linked to the diagnosis and the expected outcome, avoiding generic or out-of-context prescriptions. When an intervention includes multiple actions, it can be associated with more than one NIC intervention, as long as the correlation is justifiable. If any intervention cannot be mapped, document and justify the identified gap. Ensure that all used interventions are described in the annex “List of Nursing Interventions (NIC).” 5. Response Format The final answer must be displayed in a structured chart containing the following columns: Clinical indicator (source: Manual of Diagnosis and Management of Type 2 Diabetes (HEARTS-D)) Nursing diagnoses Nursing outcomes Nursing interventions

The GPT-4® version was used. PDF files of the manual for Diagnosis and Management of Type 2 Diabetes (HEARTS-D) were attached, along with a list describing the NANDA-I ND, including their diagnostic indicators, a list of the NIC NI, and a list of the NOC NO. The command used in the prompt was also included. Initially, exploratory tests were conducted with different versions of the prompt until a more precise and optimized formulation was achieved. After executing the first prompt command (see Table 1), which aimed to identify the clinical indicators contained in the Diagnostic and Management of Type 2 Diabetes manual (HEARTS-D), a manual review was conducted, with direct verification in the PAHO document itself. This review was necessary to prevent the cross-mapping performed by the AI from including ND, NO, and NI that were not relevant to the clinical context presented in the PAHO manual (see Table 2). This precaution aimed to ensure the accuracy, relevance, and applicability of the analysis developed. Only after this analysis was it possible to safely proceed to the next stage of the cross-mapping process.

In the second stage, the second prompt command was executed (see Table 1), now with the attachment of the lists of ND (NANDA-I - 13^th^ edition),^10^ NO (NOC - 7th edition),^11^ and NI^12^ (NIC - 7^th^ edition). This command aimed to perform cross-mapping between the previously identified clinical indicators and the elements of the three nursing classifications (see Table 3). Next, a complementary manual mapping stage was carried out to ensure compatibility with the current edition of the classifications used, as well as to address clinical nuances that might not be fully captured by AI (see Table 4). This comparison between the AI results and the human analysis allowed for a more robust evaluation, ensuring greater reliability and clinical applicability for the final mapping. The manual mapping was carried out independently by two researchers in this study, and any discrepancies were resolved with the involvement of a third researcher.

### Data Collection and Organization

Data collection was carried out during May 2025. The results were generated by AI and organized in a table.

## Results

In the first command, the AI (GPT-4®) identified a total of 43 clinical indicators related to diabetes. After a manual review, 30 valid clinical indicators were confirmed. Of these, 23 showed agreement between the two identification methods (AI mapping versus manual mapping). See Table 2.

**Table 2:** Comparative table of the identification of clinical indicators of diabetes based on the HEARTS-D manual: AI versus manual review

Clinical indicators by AI	Clinical indicators by manual review
Excessive thirst Frequent urination Blurred vision Fatigue Unintentional weight loss Severe dehydration Kussmaul breathing Vomiting Altered level of consciousness Coronary artery disease Stroke Kidney disease Vision loss Diabetic foot Overweight and obesity Sedentary lifestyle Advanced age Family history (first-degree relatives) History of gestational diabetes Cardiovascular disease and its risk factors Ethnicity: Asian (South Asia), Afro-Caribbean, and Hispanic. Elderly Pregnant women > 22 years old History of gestational diabetes Family history of diabetes Unfavorable socioeconomic conditions African descent Exposed to prolonged fasting Period of rapid growth Diabetes mellitus Cardiovascular diseases Kidney, liver, and pancreatic diseases Hypertension Cerebrovascular disorders Peripheral neuropathy Diabetic retinopathy Infections and ulcers in the lower limbs Polycystic ovary syndrome Mental disorders Pharmaceutical preparations (metformin, gliclazide, and insulin) Monitoring with HbA1c and fasting blood glucose Use of statins and antiplatelet agents Renal therapies (dialysis or transplant in advanced cases)	Excessive thirst Frequent urination Blurred vision Fatigue Unintentional weight loss Signs of severe dehydration Kussmaul breathing Vomiting Altered level of consciousness Coronary artery disease Stroke Kidney disease Vision loss Diabetic foot Overweight and obesity Sedentary lifestyle Advanced age Family history (first-degree relatives) History of gestational diabetes Cardiovascular disease and associated risk factors Ethnicity (South Asian, Afro-Caribbean, and Hispanic) Hypoglycemia Diabetic ketoacidosis (DKA) Hyperosmolar Hyperglycemic State (HHS) Peripheral or autonomic neuropathy Diabetic retinopathy Diabetic nephropathy Coronary heart disease Cerebrovascular disease Peripheral vascular disease

Based on the mapping carried out with the support of AI, 30 ND in NANDA-I, 30 NO in NOC, and 30 NI in NIC were identified. These results were organized in the table below to highlight the links established between the clinical indicators extracted from the HEARTS-D manual and the nursing classifications. See Table 3.

**Table 3:** Cross-mapping of nursing diagnoses (NANDA-I), nursing outcomes (NOC), and nursing interventions (NIC), based on the HEARTS-D manual: AI-generated

CLINICAL INDICATORS	NURSING DIAGNOSES (NANDA-I)	NURSING OUTCOMES (NOC)	NURSING INTERVENTIONS (NIC)
Excessive thirst Frequent urination Blurred vision Fatigue Unintentional weight loss Signs of severe dehydration Kussmaul breathing Vomiting Altered level of consciousness Acute coronary disease Stroke Kidney disease Vision loss Diabetic foot Overweight and obesity Sedentary lifestyle Advanced age Family history (first-degree relatives) History of gestational diabetes Cardiovascular disease and associated risk factors Ethnicity (South Asian, Afro-Caribbean, and Hispanic) Hypoglycemia Diabetic ketoacidosis (DKA) Hyperosmolar hyperglycemic state (HHS) Peripheral or autonomic neuropathy Diabetic retinopathy Diabetic nephropathy Coronary heart disease Cerebrovascular disease Peripheral vascular disease	Fluid volume deficit	Hydration	Fluid monitoring
	Impaired urinary elimination	Urinary elimination	Urinary elimination control
	Visual impairment	Vision status	Prevention of visual injury
	Fatigue	Energy level	Energy conservation
	Unbalanced nutrition: less than bodily needs	Nutritional status	Nutrition management
	Nausea	Nausea and vomiting control	Nausea control
	Risk of acute confusion	Neurological status	Monitoring of consciousness
	Ineffective tissue perfusion - cardiac	Cardiac tissue perfusion	Cardiac care
	Risk of impaired skin integrity	Tissue integrity: skin and mucous membranes	Foot care
	Excessive sedentary behaviors	Physical fitness	Encouragement of activity
	Risk of fall	Prevention of falls	Prevention of falls
	Risk of type 2 diabetes	Prevention of diabetes	Nutritional counseling
	Risk of unstable blood sugar	Hydration	Blood sugar control
	Risk of injury	Prevention of injuries	Neurovascular monitoring
	Impaired gas exchange	Renal perfusion	Renal failure care
	Ineffective cerebral blood flow	Neurological status	Seizure precautions
	Ineffective tissue perfusion	Peripheral tissue perfusion	Peripheral circulation care

Subsequently, a manual cross-mapping of the 30 validated clinical indicators was carried out based on the PAHO manual. For this stage, a list with ND, NO, and NI extracted from the NANDA-I, NOC, and NIC classifications was used, following the same correspondence criteria adopted in the automated stage, according to the cross-mapping proposal by Lucena and Barros.^8^ See Table 4.

**Table 4:** Cross-mapping of nursing diagnoses (NANDA-I), nursing outcomes (NOC), and nursing interventions (NIC), based on the HEARTS-D manual: performed manually

Clinical indicators	Nursing diagnoses (NANDA-I)	Nursing outcomes (NOC)	Nursing interventions (NIC)
Sede excessiva Frequent urination Blurred vision Fatigue Unintentional weight loss Signs of severe dehydration Kussmaul breathing Vomiting Altered level of consciousness Acute coronary disease Stroke Kidney disease Vision loss Diabetic foot Overweight and obesity Sedentary lifestyle Advanced age Family history (first-degree relatives) History of gestational diabetes Cardiovascular disease and associated risk factors Ethnicity (South Asian, Afro-Caribbean, and Hispanic) Hypoglycemia Diabetic ketoacidosis (DKA) Hyperosmolar hyperglycemic state (HHS) Peripheral or autonomic neuropathy Diabetic retinopathy Diabetic nephropathy Coronary heart disease Cerebrovascular disease Peripheral vascular disease	Excessive fatigue load	Fatigue level	Energy control
	Ineffective self-management of nausea	Nausea and vomiting control	Vomiting control
	Risk of acute confusion	Delirium level	Neurological monitoring
	Inadequate nutritional intake	Nutritional status	Nutritional counseling
	Impaired gas exchange	Respiratory function: gas exchange	Acid-base control: metabolic acidosis
	Ineffective self-management of health	Risk control	Education: disease process
	Risk of impaired skin integrity	Tissue integrity: skin and mucous membranes	Foot care
	Excessive sedentary behaviors	Physical fitness	Exercise promotion
	Risk of ineffective self-management of blood glucose levels	Risk control	Education: disease process
	Risk of falls in adults	Fall prevention behavior	Fall prevention
	Risk of physical injury	Risk detection	Injury care
	Ineffective peripheral tissue perfusion	Tissue perfusion: peripheral	Circulatory precautions
	Inadequate fluid volume	Fluid volume balance	Fluid and electrolyte volume control
	Impaired urinary elimination	Urinary continence	Urinary continence care
	Ineffective breathing pattern	Respiratory status	Airway control

## Discussion

The use of AI in cross-mapping clinical indicators from the HEARTS-D manual with the NANDA-I, NOC, and NIC classifications demonstrated significant potential to streamline and standardize the care process. The creation of the prompt was a central methodological element for the quality of the cross-mapping. Prompt engineering was conducted interactively, with successive adjustments until an effective hierarchical structure was achieved, capable of guiding the AI from the extraction of indicators to their association with ND, NO, and NI, ensuring clarity and coherence throughout the process. The inclusion of rules for semantic matching allowed the identification of conceptually compatible ND, even with terminological variations. This process reduced ambiguities and model “hallucinations,” ensuring greater consistency in the findings, in line with studies highlighting the importance of prompt engineering strategies in educational and clinical contexts.^6^^,^^9^ The possibility of reproducing and applying this prompt in different care settings represents an advancement, as it expands the potential for standardization and comparability of data, in addition to supporting the development of personalized care protocols.^6^^,^^7^^,^^9^

Although AI (GPT-4®) has demonstrated agility and standardization in cross-mapping, the study showed that human clinical judgment remains essential. The manual review of the 43 clinical indicators initially identified by AI resulted in the validation of 30, with 23 of these indicators showing agreement between the automated and manual methods. These findings support the view that qualified professional mediation is crucial for the effectiveness of AI in nursing, highlighting the importance of integrating technology with human reasoning.^6^ Manual mapping proved to be more sensitive to clinical nuances, correcting inconsistencies, adjusting outdated classification nomenclature, and identifying additional diagnoses not suggested by AI. This interpretive ability shows that, although AI can recognize general patterns, the personalization of care still relies on the nurse's critical analysis.^6^^,^^13^

AI still faces challenges in semantic distinction,^13^ which may compromise the individualization of the care plan. Among the identified ND, “Inadequate nutritional intake” and “Risk of ineffective self-management of blood glucose” stand out, recognized as central in the care of diabetic patients. Although initially suggested by AI in outdated versions of NANDA-I, their manual validation confirmed their clinical relevance. These ND are widely described in the literature as central to the care of diabetic patients, whose metabolic condition imposes a high risk of complications associated with poor nutrition and glycemic imbalance.^14^ Other ND mapped as “Impaired skin integrity risk” and “Ineffective peripheral tissue perfusion” are directly related to the prevention of complications such as diabetic ulcers and amputations. In addition, the ND “Ineffective health self-management” and “Risk of ineffective self-management of blood glucose” reflect the growing concern with self-care and therapeutic adherence, considered one of the main challenges in primary care for diabetes.^15^

Among the most common symptoms of diabetic ketoacidosis (DKA) are nausea, vomiting, and difficulty breathing, as described by the American Diabetes Association. These clinical signs directly correspond to the defining characteristics of the ND “Ineffective nausea control” and “Ineffective breathing pattern,” both identified in the mapping. The presence of these diagnoses underscores the importance of early recognition of DKA symptoms by nursing staff, promoting timely interventions and the prevention of serious complications associated with metabolic decompensation. Fatigue also appears as a multifactorial symptom in patients with diabetes, related to chronic hyperglycemia, systemic inflammation, and emotional stress.^14^ The diagnosis “Excessive sedentary behavior” also appears in literature reviews on diabetes, highlighting the impact of a sedentary lifestyle on insulin resistance and metabolic control.^14^

In turn, the manual review highlighted diagnoses that were more sensitive to the sociocultural context, such as those related to fall risk and skin integrity, with a strong impact on clinical outcomes in patients with diabetic complications. The ND “Impaired urinary elimination” and “Risk of falls in adults” were also mentioned in a review that compiles evidence on ND in patients with diabetes mellitus.^14^ The presence of urinary changes, such as dysuria, hesitation, incontinence, and nocturia, can significantly compromise the patient's quality of life and autonomy. Associated with this, the 'Risk of falls' becomes relevant in light of the common neurological complications in diabetic patients, such as peripheral neuropathy, which affects sensation and balance.^17^ These findings underscore the need for integrated nursing care to prevent complications and promote greater patient safety.

Regarding the NO mapped in this study, it was observed that they are also described in the literature for patients with diabetes. The NO “Nutritional status,” “Respiratory status: gas exchange,” and “Level of acute confusion” are widely recommended as measurable outcomes for monitoring these patients.^15^^,^^18^ The NO “Risk control” associated with the ND “Ineffective self-management of blood glucose pattern” stands out as one of the main indicators in managing the self-sufficiency of diabetic patients.^15^ In this context, the use of the NOC classification promotes the standardization of care, the evaluation of its effectiveness, and interprofessional communication.^19^ Regarding NI, the proposed actions - such as nutritional counseling, foot care, and promotion of exercise - are aligned with practices proven effective in preventing chronic complications and improving patients' quality of life.^16^^,^^20^ In this sense, AI can contribute to the systematization and standardization of prescriptions, provided its implementation is accompanied by clinical validation strategies based on the best available evidence.

The simultaneous use of the NANDA-I, NOC, and NIC nursing classifications in the present study may ensure the interoperability of information and the systematization of care. This integration of standardized languages allows for the logical organization of clinical data, the standardization of records, and the development of care plans aligned with patients’ needs.^21^ AI has great potential to standardize care and optimize professionals’ time, but its effective implementation requires active collaboration from nurses in the development of the tools. Clinical judgment remains essential in complex cases and must be recognized as an indispensable criterion for validating the results generated by clinical decision support technologies.^(3, 21)^

Conclusion. This research highlighted the potential of AI, represented by the GPT-4® model, as a tool to support cross-mapping between clinical indicators from the HEARTS-D manual and the nursing classifications NANDA-I, NOC, and NIC. AI demonstrated agility in identifying and organizing data, optimizing the analysis process. However, human clinical judgment was indispensable to validate and refine the results, correcting inconsistencies and capturing clinical nuances not recognized by AI. The integration of AI and clinical reasoning can contribute to the systematization of care, the development of evidence-based protocols, and the improvement of outcomes in patients with diabetes.

As a limitation of the study, the absence of empirical validation of the mapped ND, NO, and NI is highlighted, which restricts the direct application of the findings in clinical practice. Future studies could expand on this proposal by conducting clinical validation of the findings, applying the methodology to other clinical conditions, and exploring the incorporation of AI into electronic health systems.

## References

[1] 1. World Health Organization. Diabetes [Internet]. World Health Organization. 2024. Available from: https://www.who.int/news-room/fact-sheets/detail/diabetes

[2] 2. Conselho Federal de Enfermagem (BR). Resolução nº 736, de 17 de janeiro de 2024. Dispõe sobre a implementação do Processo de Enfermagem em todo contexto socioambiental onde ocorre o cuidado de enfermagem [Internet]. Brasília, DF: COFEN; 2024. Available from: https://www.cofen.gov.br/resolucao-cofen-no-736-de-17-de-janeiro-de-2024/

[3] Zhang T, Wu X, Peng G, Zhang Q, Chen L, Cai Z (2021). Effectiveness of standardized nursing terminologies for nursing practice and healthcare outcomes: A systematic review. International Journal of Nursing Knowledge.

[4] 4. Diagnóstico e manejo do diabetes tipo 2 (HEARTS-D) [Internet]. Paho.org. 2020. Available from: https://www.paho.org/pt/documentos/diagnostico-e-manejo-do-diabetes-tipo-2-hearts-d

[5] Rodrigues JAP, Lacerda MR, Galvão CM, Cubas MR (2022). Use of the International Classification for Nursing Practice in the construction of a care protocol. Revista Brasileira de Enfermagem.

[6] Karacan E (2024). Evaluating the Quality of Postpartum Hemorrhage Nursing Care Plans Generated by Artificial Intelligence Models. Journal of Nursing Care and Quality.

[7] McGrow K (2025). Artificial intelligence in nursing: A journey from data to wisdom. Nursing.

[8] Lucena A de F, Barros ALBL de (2005). Mapeamento cruzado: uma alternativa para a análise de dados em enfermagem. Acta Paulista de Enfermagem.

[9] Diyab A, Frost RM, Fedoruk BD, Diyab A (2025). Engineered prompts in ChatGPT for educational assessment in software engineering and computer science. Education Sciences.

[10] Heather HT, Kamitsuru S, Lopes CT (2024). Diagnósticos de Enfermagem da NANDA-I. Artmed Editora.

[11] Moorhead S, Johnson M, Massone M, Swanson E (2024). NOC Classificação dos Resultados de Enfermagem. Grupo GEN.

[12] Butcher HK, Bulechek GM, Dochermann JM, Wagner CM (2020). NIC - Classificação das Intervenções de Enfermagem. Grupo GEN.

[13] O'Connor S, Peltonen LM, Topaz M, Chen LA, Michalowski M, Ronquillo C, Stiglic G, Chu CH, Hui V, Denis-Lalonde D (2024). Prompt engineering when using generative AI in nursing education. Nurse Education and Practice.

[14] Serra EB, Ferreira AGN, Pascoal LM, Rolim ILTP (2020). Nursing diagnoses in diabetic patients: an integrative review. Revista Enfermagem UERJ.

[15] Garizábalo-Dávila CM, Cañon-Montañez W, Rodríguez-Acelas AL (2024). Nursing outcomes and social support intervention for diabetes self-management: consensus study. Revista Cuidarte.

[16] 16. American Diabetes Association. Diabetes & DKA (Ketoacidosis) | ADA [Internet]. diabetes.org. 2024. Available from: https://diabetes.org/about-diabetes/complications/ketoacidosis-dka/dka-ketoacidosis-ketones

[17] Carlos AG, Dias V da N, Perracini MR, Doná F, Sousa AGP, Gazzola JM (2024). Equilíbrio postural e fatores associados ao risco de quedas em idosos com diabetes mellitus tipo 2. Revista Brasileira de Geriatria y Gerontolologia.

[18] Diniz FS, Rodrigues JA, Vaez AC, Santos AD, Araújo D da C, Silva B de A, de Andrade JS, Sousa PHSF (2021). Plano de cuidado de enfermagem para usuários com diabetes Mellitus. Brazilian Journal of Development.

[19] Rodríguez-Suárez CA, González-de la Torre H, Hernández-De Luis MN, Fernández-Gutiérrez DÁ, Martínez-Alberto CE, Brito-Brito PR (2023). Effectiveness of a Standardized Nursing Process Using NANDA International, Nursing Interventions Classification and Nursing Outcome Classification Terminologies: A Systematic Review. Healthcare (Basel).

[20] Aríztegui Echenique AM, San Martín Rodríguez L, Marín Fernández B (2020). [Effectiveness of nursing interventions in the control of type 2 diabetes mellitus]. Anales del Sistema Sanitario de Navarra.

[21] Fennelly O, Grogan L, Reed A, Hardiker NR (2021). Use of standardized terminologies in clinical practice: A scoping review. International Journal of Medical Informatics.

